# Knowledge of Latin American Obstetricians and Gynecologists regarding Heavy Menstrual Bleeding

**DOI:** 10.1155/2016/6870679

**Published:** 2016-08-25

**Authors:** Luis Bahamondes, Victor Marin, Silvia Ciarmatori, Agnaldo L. Silva-Filho, Juan Manuel Acuña, Maria Y. Makuch

**Affiliations:** ^1^Department of Obstetrics and Gynecology, University of Campinas Medical School (UNICAMP), Campinas, SP, Brazil; ^2^Bayer HealthCare, Mexico City, Mexico; ^3^Hospital Italiano, Buenos Aires, Argentina; ^4^Federal University of Minas Gerais, Belo Horizonte, MG, Brazil; ^5^Florida International University College of Medicine, Miami, Fl, USA

## Abstract

*Background*. Heavy menstrual bleeding (HMB) is a common gynecological complaint affecting quality of life.* Objectives*. To assess knowledge on diagnosis and treatments of HMB of Latin American (LA) obstetricians and gynecologists (OBGYNs).* Methods*. A survey was conducted during a scientific meeting, organized to provide updated information on topics of reproductive medicine to OBGYNs from 12 LA countries who were invited to respond to a multiple-choice questionnaire.* Results*. Of the 210 OBGYNs participating in the survey, from 169 (80.4%) to 203 (96.7%) answered the questions. Most respondents (80%) gave accurate answers regarding the amount of blood loss which defines HMB, underreported the proportion of women who consulted due to HMB, and were aware that the use of combined oral contraceptives (COCs) with ethynyl estradiol is not an adequate treatment in women with HMB. Female OBGYNs and those who worked in the private sector were more prone to report a higher possibility of improvement of HMB with a COC that contained estradiol valerate and dienogest or with a levonorgestrel-releasing intrauterine system.* Conclusions*. In general, the respondents were aware of the importance of HMB in gynecological practice and of the new medical treatments and underreported the proportion of women who consulted due to HMB.

## 1. Introduction

Menstrual irregularities are a common complaint in gynecological consultations and heavy menstrual bleeding (HMB) is one of the most common menstrual irregularities reported by women. HMB has a significant deleterious effect on women's quality of life (QofL) and in general increases the number of consultations and treatments required, consequently increasing costs of healthcare systems [1]. The worldwide accepted definition of HMB is a blood loss of >80 mL per cycle [2, 3]; however, the possibility of measuring the exact amount of blood loss is restricted to research studies due to the complexity of the techniques [4]. Consequently, in clinical practice, HMB is defined as “*excessive menstrual blood loss which interferes with the woman's physical, emotional, social, and material QofL and which can occur alone or in combination with other symptoms*” [5].

It has been estimated that almost 18.5% of gynecological visits in the US and 20% in the UK are due to HMB [6]. Furthermore, in most of the women with HMB, it is not possible to detect any structural or organic pathology and even when an endometrial, uterine, or endocrine abnormal cause is detected, HMB is poorly understood [7].

HMB is a gynecological event that only occurs in women at reproductive age and consequently many of those women who need control of the menstrual irregularity also need contraception. Due to the complexity of the diagnosis and etiology, at present, therapies for HMB are still under debate and many still present poor improvement of the medical condition [7]. The goal of treatments for HMB is to reduce menstrual flow and improve QofL. Many women want to preserve intact uterus due to fertility desire; conservative medical treatments are frequently preferable to surgical procedures [8–10].

In most of the Latin American (LA) countries, obstetricians and gynecologists (OBGYNs) are the healthcare professionals (HCPs) who are the first line of attention in women's health and the main cadre to provide information and attention on placement and prescription of contraceptives. For the reasons above, it is important that OBGYNs are updated on the importance of HMB within the context of women's health, its diagnosis, and treatments in order to provide adequate counseling and treatment. According to these considerations, the aim of this report was to assess some knowledge about diagnosis and treatments of HMB of a group of LA OBGYNs.

## 2. Material and Methods

This is the third publication of the data obtained through a multiple-choice questionnaire completed by LA OBGYNs using an Interactive Audience Systems Response on occasion of a scientific meeting in Chile organized by Bayer HealthCare in the last quarter of 2014 [11, 12]. The OBGYNs came from 12 countries (Argentina, Bolivia, Brazil, Chile, Colombia, Costa Rica, Ecuador, Guatemala, Mexico, Paraguay, Peru, and Venezuela). The objective of the meeting was to discuss updated information on selected themes of women's reproductive health related to the use of hormonal and nonhormonal contraceptive methods and HMB. Data on knowledge and attitudes regarding unplanned pregnancies and combined oral contraceptives (COCs) and intrauterine contraceptives (IUCs) use has already been published [11, 12]. For this paper, questions related to HMB and related health issues were selected.

All the OBGYNs in this meeting had experience in providing attention to reproductive health and contraception in their everyday clinical practice. At the time of registration for the meeting, all the OBGYNs were invited to take part in the study and all participants received an explanation of the objectives of the survey, of the anonymity since the keypads were only identified with a number, and of the voluntariness of participation. Those who accepted received electronic keypads to answer the questions regarding themes discussed after each lecture and by answering the questions through the electronic device the OBGYNs were giving their consent to participate in the study.

The information provided about the survey and consent of participation and the distribution and collection of the electronic devices were supervised by one of the researchers to guarantee the accuracy and uniformity of the procedure. The study protocol and the procedure for obtaining consent were approved by the Ethical Committee of the University of Campinas (UNICAMP), Brazil.

The questionnaire, designed especially for this survey, contained questions regarding the demographic information (nationality, age, gender, clinical experience in reproductive health, and site/location of work (public, private, or both)) of the participants and regarding the knowledge and practice of the OBGYNs about HMB and health related issues. The questions were multiple-choice. For each theme discussed during the meeting, five questions were developed with five possible answer choices for each question. The questions were developed by the researchers who conducted this study and discussed via e-mail with a group of LA OBGYNs of recognized expertise in the area of HMB who did not participate in the meeting. To establish the correct answer for each question, the group who developed the questionnaire took into account the LA reality and the treatments used in the region. Participants had ~3 minutes after each presentation to answer the questions and after that the system automatically locked.

For statistical analysis, the dependent variables were analyzed taking into account age (≤49, ≥50 years old), gender (male/female), and the sector in which the OBGYN worked (exclusively public, both private and public). The statistical tests used were Pearson *χ* ^2^, Yates *χ* ^2^, and Fisher-exact tests. The software used was SPSS v.20.0. The significance was established at *P* < 0.05.

## 3. Results

From the total of 210 OBGYNs participating in the meeting, responses to the questions on HMB ranged from 169 (80.4%) to 203 (96.7%) responses. The mean (± standard deviation, SD) age of the participants was 48.7 ± 10.6 (range 30 to 72); 122 (58.1%) were male and 88 (41.9%) were female. Additionally, 112 (55.3%) reported practicing in both the public and the private sectors and 81 (38.6%) only in the private sector.

The answers of the participating OBGYNs regarding knowledge on some aspects of HMB are presented in Table 1. Eighty percent of the respondents were aware of the amount of blood loss which defines HMB; however, in general, they were less aware of the proportion of women who consulted due to HMB. Almost one-third identified endometrial hyperplasia as a separate structural abnormality associated with HMB, and two-thirds of the respondents answered that HMB is an infrequent and subjective pathology.

Information regarding treatment of HMB is presented in Table 2. The respondents were aware that the use of COC with ethynyl estradiol (EE) does not provide adequate relief of bleeding in women with HMB, although some of them (between one quarter and one-third) reported higher possibility of improvement of HMB using a COC with estradiol valerate and dienogest (E_2_Val/DNG) or with the placement of a levonorgestrel-releasing intrauterine system (LNG-IUS).

## 4. Discussion

The findings of our study showed that this group of LA OBGYNs presented some misconceptions regarding different specific aspects of HMB. It is worthwhile to note that some misconceptions persisted among some of the participants. We observed, in our study, that almost one-third of the respondents reported that the percentage of women who consulted for HMB represented more than 20% of the consultations. This reported percentage was higher than those reported in previous studies [6]. These figures may be expected if we take into account the total proportion of women who consulted due to menstrual bleeding disturbances, but not for consultation regarding HMB [1, 6]. However, it is important to observe that the participant OBGYNs were aware of the amount of blood loss to diagnose HMB, although one-third were concerned that the bleeding amount cannot be measured properly.

Furthermore, more than 50% answered that the disorder is infrequent. This result is contradictory to previous results that described that HMB is one of the most common complaints during consultation among women who consulted due to menstrual abnormalities [6]. It has been described that HMB has significant effects on women's quality of life (QofL) [1, 6, 7, 13]. In a Swedish-based study [13] with 1547 women at reproductive age, the authors evaluated QofL using the instrument SF-36 in a community-based cross-sectional web-based questionnaire. The results showed that 32% of the interviewed women presented HMB. Women who complained of HMB were associated with limited social and professional activities as well as negative perceptions and significantly worse health related QofL when compared with women with normal menstrual patterns in all domains of the questionnaire. Awareness of OBGYN regarding HMB is an important issue because early diagnosis and treatment of this disorder as well as bleeding control could have an important influence on QofL in this group of women.

HMB is recognized as a worldwide benign, debilitating, challenging health and social pathology which can cause iron deficiency. As an example in the United Kingdom, it affects 20–25% of the women at reproductive age which means that millions of women have either anemia or iron storage deficiency [14–17]. It is estimated that more than 60% of women with HMB and blood loss greater than 80 mL have anemia and the incidence increases if blood loss exceeds 80 mL. It is worthwhile to take into account the fact that it has been described that many women may complain of HMB and present normal hemoglobin level [18]. Due to the fact that HMB does not necessarily correlate with anemia, this could be a possible explanation as to why some women take a long time before consulting and after consulting in receiving treatment. Consequently, it is important that OBGYNs become aware of the importance of HMB in clinical reproductive health practice (with or without anemia) and not only aware of the symptoms but also aware of the complexity of the diagnosis and the fact that HMB may affect women's QofL [6, 7].

The OBGYNs participating in our study were less aware of the abnormalities associated with HMB and almost one-third answered that endometrial hyperplasia is a nonstructural abnormality associated with HMB. It was described and approved by the International Federation of Gynecology and Obstetrics (FIGO) Executive Board [19] that “structural abnormalities associated with abnormal uterine bleeding (AUB) included the PALM-COEIN nomenclature (polyp; adenomyosis; leiomyoma; malignancy and hyperplasia; coagulopathy; ovulatory dysfunction; endometrial; iatrogenic; and not yet classified)” and as observed hyperplasia was included with structural abnormalities.

The use of combined oral contraceptives (COCs) is associated with several health benefits and one of them is the transformation of normal endometrium on a thinner tissue and consequently the potential effect of treating HMB [20]. The OBGYNs who participated in our study were aware that the use of monophasic COCs with EE in a 21/7 regimen presented low effectiveness in the treatment of women with HMB. COCs are in use worldwide to treat women with acute and chronic HMB, despite the lack of reliable information from a randomized clinical trial (RCT) to support the use [21]. Additionally, Matteson and coworkers recommended the use of LNG-IUS over COCs, luteal phase progestogens, and nonsteroidal anti-inflammatory drugs [22]. Presently, there is limited evidence showing that regular intake of COCs could provide cycle control and could reduce menstrual blood loss. Furthermore, due to the fact that COCs also provide contraception, this could be an acceptable therapy for women with menstrual disorders [21].

A Cochrane Collaboration review [23] of COCs use for HMB found only one RCT which met the inclusion criteria. This RCT, a crossover study of mefenamic acid, low-dose danazol, naproxen, and a COC (30 *μ*g EE and 150 *μ*g levonorgestrel (LNG) in a 21/7 regimen), showed similar results in bleeding control among the various agents tested on reduction of blood loss [24]. Moreover, in a recent review [20], COCs were effective in HMB control; however, the effectiveness was similar to long-term progestin therapy, superior to short-term progestin therapy, and less effective than the LNG-IUS. Another therapy in use to treat HMB is oral tranexamic acid which was reported to be superior to placebo, mefenamic acid, and progestogen during the luteal phase [21]. In a double-blind placebo-controlled RCT with oral tranexamic acid (3.9 g/day for 5 days over 6 cycles), women in the treatment arm presented significant improvement in blood loss (40.4%) compared with placebo (8.2%) and improving of QofL [25].

The participants of our survey were aware of the effectiveness of the new COC containing the newer E_2_Val/DNG regimen. Scientific evidence has proved that such COC is highly effective in reducing HMB. A study which compared a COC containing E_2_V/DNG with a COC with EE/LNG for treatment of HMB showed that women using the E_2_V/DNG COC presented significantly shorter and lighter uterine bleeding and fewer bleeding and spotting days than users of EE/LNG pills [26]. Moreover, it was observed that this preparation significantly reduced HMB compared to placebo [27, 28]. Although many women who use COCs can expect a reduction in the volume of uterine bleeding, physicians must be aware that different COCs have different effects upon menstrual cycle bleeding control.

Finally, the participants were aware that one of the noncontraceptive benefits of the LNG-IUS is HMB control. It was described that up to 60% of the users presented improvement in blood loss, hemoglobin, and iron stores and reduction of anemia up to one year [4, 29]. Furthermore, women who presented objectively HMB (pretreatment blood losses between 80 mL and 400 mL) showed a reduction in uterine bleeding of 98% at one year after placement of an LNG-IUS and no woman had an objective blood loss >20 mL after that period of treatment [30]. Due to the body of evidence about the use of an LNG-IUS in women suffering from HMB, the device is approved in more than 100 countries for this purpose and its efficacy is equal or superior to other therapies [31]. Consequently, LNG-IUS is the first line of medical treatment for nonorganic HMB in several national and international guidelines [5] and many HCPs consider the LNG-IUS a first choice for women with HMB with or without any pathology, with hemostatic disorders, or among women with uterine fibroids [32].

The study has some limitations. We have information that all participants had clinical practice in reproductive health including contraception, but we did not obtain the scientific profile of the participants or information about work in academic institutions. The answers they provided could be influenced by a courtesy bias, mainly the responses regarding COC and the LNG-IUS, because both products are manufactured by the sponsor of the meeting. Furthermore, due to the fact that the answers were provided after each lecture, it could be legitimate to suppose that our results are based on the participating OBGYNs' previous training and not necessarily on baseline attitudes or reaction after each lecture. Also, it has to be taken into account that the sample size was not estimated in a scientific manner but it was a convenience sample according to the number of participants in the meeting.

It is important to take into account the fact that HMB is a gynecological medical condition which affects many women and requires an understanding of the etiology, diagnosis, underlying pathologies, and treatments. OBGYNs and other HCPs must be well capacitated in order to offer to their patients the best treatments. In conclusion, in this study, we observed that there was awareness regarding the importance of HMB in clinical practice; however, some misunderstandings in terms of knowledge of treatments were identified among LA OBGYNs. This information is important to elaborate strategies and to train and maintain continuous updated information for LA OBGYN. The fact that many of the participants reported that HMB is an infrequent and subjective disorder could highlight the need for better clinical tools for its recognition by women and HCPs as well as for its diagnosis.

## Figures and Tables

**Table 1:** Answers provided by the OBGYNs regarding some aspects of heavy menstrual bleeding (HMB).

	Age (%)		Gender (%)		Working in (%)	
	≤49 ys	≥50 ys	Female	Male	Public sector	Private/public sector
*What is the proportion of women who consult due to HMB? (n = 183) *						
<5–19%	62.5	60.4	58.1	66.1	67.6	60.0
20–30%	37.5	39.6	41.9	33.9	32.4	40.0
*What are your criteria for diagnosis of HMB? (n = 169)*						
Bleeding 30–75 mL	16.3	30.0	14.9	30.9	32.4	19.1
Bleeding >80 mL	83.7	70.0	85.1	69.1	67.6	80.9
*The non-structural abnormalities associated to HMB are, except for (n = 173)*						
Hematological disorders, ovulatory disfunction, iatrogenic	60.2	74.4	65.7	70.0	68.8	67.9
Endometrial hyperplasia	39.8	25.6	34.3	30.0	31.2	32.1
*In your opinion what are the major challenges to diagnose HMB at office level? (n = 187)*						
In clinical practice it is not possible to measure the amount of blood loss	35.8	37.5	38.5	35.8	41.7	33.9
The pathology is infrequent and subjective	64.2	62.5	61.5	64.2	58.3	66.1

**Table 2:** Answers provided by the OBGYNs regarding some therapeutic aspects of heavy menstrual bleeding (HMB).

	Age (%)		Gender (%)		Working in (%)	
	≤49 ys	≥50 ys	Female	Male	Public sector	Private/public sector
*What is the proportion of women with improvement of HMB with a COC with EE? (n = 203) *						
<50%	98.0	79.4	87.1	89.7	86.7	90.0
Up to 80%	2.0	20.6	12.9	10.3	13.3	10.0
*What is the proportion of women with improvement of HMB with a COC with estradiol Val/DNG? (n = 199) *						
30–40%	27.7	39.0	27.8	37.7	38.8	29.3
41–79%	41.2	28.4	28.3	35.2	32.3	33.6
>80%	31.1	32.6	43.9	27.1	28.9	37.1
*What is the proportion of women with improvement of HMB with an LNG-IUS at the end of the first year of use? (n = 201)*						
30–79%	75.6	75.6	82.9	70.2	81.6	71.7
>80%	24.4	24.4	17.1	29.8	18.4	28.3

OBGYNs: obstetricians and gynecologists; COC: combined oral contraceptive; EE: ethynyl estradiol; LNG-IUS: levonorgestrel-releasing intrauterine system.

## References

[1] Nicholson W. K., Ellison S. A., Grason H., Powe N. R. (2001). Patterns of ambulatory care use for gynecologic conditions: A National Study. *American Journal of Obstetrics and Gynecology*.

[2] Coulter A., Bradlow J., Agass M., Martin-Bates C., Tulloch A. (1991). Outcomes of referrals to gynaecology outpatient clinics for menstrual problems: an audit of general practice records. *British Journal of Obstetrics and Gynaecology*.

[3] Bahamondes L., Ali M. (2015). Recent advances in managing and understanding menstrual disorders. *F1000Prime Reports*.

[4] Ragni M. V., Machin N., Malec L. M. (2016). Von Willebrand factor for menorrhagia: a survey and literature review. *Haemophilia*.

[5] Marjoribanks J., Lethaby A., Farquhar C. (2016). Surgery versus medical therapy for heavy menstrual bleeding. *Cochrane Database of Systematic Reviews*.

[6] Knol H. M., Mulder A. B., Bogchelman D. H., Kluin-Nelemans H. C., van der Zee A. G. J., Meijer K. (2013). The prevalence of underlying bleeding disorders in patients with heavy menstrual bleeding with and without gynecologic abnormalities. *American Journal of Obstetrics and Gynecology*.

[7] Herman M. C., Mol B. W., Bongers M. Y. (2016). Diagnosis of heavy menstrual bleeding. *Women's Health*.

[8] Hallberg L., Högdahl A. M., Nilsson L., Rybo G. (1966). Menstrual blood loss—a population study. Variation at different ages and attempts to define normality. *Acta Obstetricia et Gynecologica Scandinavica*.

[9] Kaunitz A. M., Bissonnette F., Monteiro I., Lukkari-Lax E., Muysers C., Jensen J. T. (2010). Levonorgestrel-releasing intrauterine system or medroxyprogesterone for heavy menstrual bleeding: a randomized controlled trial. *Obstetrics and Gynecology*.

[11] Bahamondes L., Lira-Plascencia J., Martin R., Marin V., Makuch M. Y. (2015). Knowledge and attitudes of Latin American gynecologists regarding unplanned pregnancy and use of combined oral contraceptives. *International Journal of Women's Health*.

[12] Bahamondes L., Makuch M. Y., Monteiro I., Marin V., Lynen R. (2015). Knowledge and attitudes of Latin American obstetricians and gynecologists regarding intrauterine contraceptives. *International Journal of Women's Health*.

[13] Karlsson T. S., Marions L. B., Edlund M. G. (2014). Heavy menstrual bleeding significantly affects quality of life. *Acta Obstetricia et Gynecologica Scandinavica*.

[14] Cohen B. J. B., Gibor Y. (1980). Anemia and menstrual blood loss. *Obstetrical and Gynecological Survey*.

[15] Fairhurst E., Pale T. L., Fidge B. D. (1977). A comparison of anaemia and storage iron deficiency in working women. *Proceedings of the Nutrition Society*.

[16] Rybo G. (1966). Clinical and experimental studies on menstrual blood loss. *Acta Obstetricia et Gynecologica Scandinavica*.

[17] Hallberg L., Högdahl A. M., Nilsson L., Rybo G. (1966). Menstrual blood loss—a population study. *Acta Obstetricia et Gynecologica Scandinavica*.

[18] Montalti C. S., Castro S., Souza-Pereira M., de Souza Medina S., Bahamondes L., Monteiro I. (2015). Anemia should not be the reason for consideration of heavy menstrual bleeding. *International Journal of Clinical Practice*.

[19] Munro M. G., Critchley H. O. D., Broder M. S., Fraser I. S. (2011). FIGO classification system (PALM-COEIN) for causes of abnormal uterine bleeding in nongravid women of reproductive age. *International Journal of Gynecology and Obstetrics*.

[20] Hoaglin D. C., Filonenko A., Glickman M. E., Wasiak R., Gidwani R. (2013). Use of mixed-treatment-comparison methods in estimating efficacy of treatments for heavy menstrual bleeding. *European Journal of Medical Research*.

[21] Bradley L. D., Gueye N.-A. (2016). The medical management of abnormal uterine bleeding in reproductive-aged women. *American Journal of Obstetrics & Gynecology*.

[22] Matteson K. A., Rahn D. D., Wheeler T. L. (2013). Nonsurgical management of heavy menstrual bleeding: a systematic review. *Obstetrics and Gynecology*.

[23] Farquhar C., Brown J. (2009). Oral contraceptive pill for heavy menstrual bleeding. *Cochrane Database of Systematic Reviews*.

[24] Fraser I. S., McCarron G. (1991). Randomized trial of 2 hormonal and 2 prostaglandin-inhibiting agents in women with a complaint of menorrhagia. *Australian and New Zealand Journal of Obstetrics and Gynaecology*.

[25] Lukes A. S., Moore K. A., Muse K. N. (2010). Tranexamic acid treatment for heavy menstrual bleeding: a randomized controlled trial. *Obstetrics and Gynecology*.

[26] Ahrendt H.-J., Makalová D., Parke S., Mellinger U., Mansour D. (2009). Bleeding pattern and cycle control with an estradiol-based oral contraceptive: a seven-cycle, randomized comparative trial of estradiol valerate/dienogest and ethinyl estradiol/levonorgestrel. *Contraception*.

[27] Fraser I. S., Parke S., Mellinger U., MacHlitt A., Serrani M., Jensen J. (2011). Effective treatment of heavy and/or prolonged menstrual bleeding without organic cause: pooled analysis of two multinational, randomised, double-blind, placebo-controlled trials of oestradiol valerate and dienogest. *European Journal of Contraception and Reproductive Health Care*.

[28] Fraser I. S., Römer T., Parke S. (2011). Effective treatment of heavy and/or prolonged menstrual bleeding with an oral contraceptive containing estradiol valerate and dienogest: a randomized, double-blind Phase III trial. *Human Reproduction*.

[29] Kaunitz A. M., Bissonnette F., Monteiro I., Lukkari-Lax E., DeSanctis Y., Jensen J. (2012). Levonorgestrel-releasing intrauterine system for heavy menstrual bleeding improves hemoglobin and ferritin levels. *Contraception*.

[30] Andersson J. K., Rybo G. (1990). Levonorgestrel-releasing intrauterine device in the treatment of menorrhagia. *British Journal of Obstetrics and Gynaecology*.

[31] Gupta J. K., Daniels J. P., Middleton L. J. (2015). A randomised controlled trial of the clinical effectiveness and cost-effectiveness of the levonorgestrel-releasing intrauterine system in primary care against standard treatment for menorrhagia: the ECLIPSE trial. *Health Technology Assessment*.

[32] Heliövaara-Peippo S., Hurskainen R., Teperi J. (2013). Quality of life and costs of levonorgestrel-releasing intrauterine system or hysterectomy in the treatment of menorrhagia: a 10-year randomized controlled trial. *American Journal of Obstetrics and Gynecology*.

